# PREDICTORS OF INSOMNIA IN INDIVIDUALS WITH MILD COGNITIVE IMPAIRMENT

**DOI:** 10.1093/geroni/igz038.1734

**Published:** 2019-11-08

**Authors:** Meghan Mattos, Eric M Davis, Carol Manning

**Affiliations:** University of Virginia, Charlottesville, Virginia, United States

## Abstract

Insomnia is a common disorder that affects up to 40% of people age 65 and older. Untreated insomnia can decrease quality of life, increase healthcare use, and exacerbate cognitive problems. Individuals with cognitive impairment experience more sleep disorders than those without cognitive concerns, yet little is known about insomnia and mild cognitive impairment (MCI). Our objective was to examine predictors of insomnia in persons with MCI (PwMCI). Using data from the National Alzheimer’s Coordinating Center Uniform Data Set, a cross-sectional study of older PwMCI was conducted. Independent sample t-tests and contingency tables with chi-square tests of independence were used to examine differences between PwMCI with and without insomnia. Multivariate binary logistic modeling was performed. The total sample (N=1543) was comprised of 234 (15.1%) with clinician-reported insomnia and 1309 (84.9%) without insomnia. PwMCI and insomnia were more likely to be younger, take more medications, and smoke cigarettes (p.05). Three variables significantly predicted insomnia in PwMCI subjects in a multivariate model: active depression (OR 1.66, 95%CI 1.21, 2.27), active anxiety (OR 2.16, 95%CI 1.57, 2.99) and arthritis (OR 1.78, 95%CI 1.33, 2.39). Differences in predictors of insomnia in PwMCI highlight the need for geriatric and mental health specialists to provide specialized care to this population. Future studies should examine conversion of PwMCI with insomnia to dementia and the compounding effects of insomnia on cognition.

